# A Novel Ultrasound Thermometry Method Based on Thermal Strain and Short and Constant Acoustic Bursts: Preliminary Study in Phantoms

**DOI:** 10.3390/s25020385

**Published:** 2025-01-10

**Authors:** Omar Gachouch, Bruno Giammarinaro, Teymour Kangot, Caterina Monini, Rémi Souchon

**Affiliations:** 1 LabTAU, INSERM, F-69003 Lyon, France; 2 Centre Léon Bérard, F-69003 Lyon, France; 3 Université Lyon 1, F-69003 Lyon, France

**Keywords:** temperature, HIFU, thermometry, therapeutic ultrasound

## Abstract

In the field of ultrasound therapy, the estimation of temperature to monitor treatments is becoming essential. We hypothesize that it is possible to measure temperature directly using a constant acoustic power burst. Under the assumption that the acoustic attenuation does not change significantly with temperature, the thermal strain induced by such bursts presents a linear relation with temperature. A mathematical demonstration is given in the introduction. Then, simulations of ultrasound waves in a canine liver model were conducted at different temperatures (from 20 °C to 90 °C). Finally, experimental measurements on phantom samples were performed over the same temperature range. The simulation and experimental results both showed a linear relation between thermal strain and temperature. This relation may suggest the foundation of a new ultrasound-based thermometry method. The potential and limitations of the method are discussed.

## 1. Introduction

Measuring the temperature of tissues to monitor and guide heat-based medical treatments such as cryotherapy, high intensity focused ultrasound (HIFU), or radiofrequency (RF) ablation is becoming essential [1,2]. Indeed, the beneficial clinical applications of HIFU or RF ablation to treat, for example, cancers in organs, have recently been reported in several investigations [3,4,5,6,7]. However, the lack of thermal information during treatments from 0 to 100 °C limits the ability to provide guidance in the assessment of a proper thermal dosimetry [8,9]. 

Nowadays, the gold standard to measure temperature changes during thermal therapy is magnetic resonance imaging (MRI) [10]. Nevertheless, the high cost and the limited availability of MRI make it unsuitable for most heating therapies. Being a low-cost and portable modality, ultrasound scanners seem to be a good alternative to offer non-invasive monitoring treatment response [1,8]. 

In the past, several methods were investigated to estimate temperature with ultrasound, usually by measuring the thermal strain, acoustic attenuation, or backscattered energy [1].

Of particular interest, thermal strain imaging is based on the detection of echo-shifts in response to thermal expansion and changes in the speed of sound (SOS) [11,12]. 

This variation in the signals measured before and after heating can be estimated in the frequency domain from a shift of the central frequency [12,13,14] or in the time domain, with the estimation of a time-delay [15,16]. To use this method, the initial temperature and speed of sound have to be known, and it requires a calibration table for the tissues being investigated. However, the estimation of the temperature is limited. In publications that explored the relation between the speed of sound and temperature in the range of 0–100 °C, the relation was parabolic for the liver, kidney, muscle, and prostate, with a maximum of around 50–70 °C [17,18,19]. Fat was an exception, exhibiting an inverted parabola [19]. The conventional thermal strain thermometry method showed promising results in the hyperthermia range (41–45 °C) [1,8] because the relation between the speed of sound and temperature is nearly linear in this range. However, its success is limited for temperatures above 50 °C, because this relation becomes parabolic [17]. The difficulty of tracking and cumulating small temperature changes over the full duration of the therapeutic procedure, along with a high sensitivity to cardiac and respiratory motion, are the main limitations of this method [11,20,21]. 

Acoustic attenuation also seems to be interesting to estimate temperature in the hyperthermia range, although attenuation is more difficult to measure than echo shift [1,8]. However, in contrast to the previous method, the sensitivity of acoustic attenuation to temperature increases above >50 °C, and becomes more interesting for high temperature ablation procedures but only by detecting coagulation rather than estimating the temperature [1,22]. 

Other studies have also shown an interest of changes in backscattered energy with temperature until 60 °C, but these variations can also be attributed to mechanical effects during HIFU treatment such as boiling or cavitation [1,23,24]. A summary of these techniques is provided in Supplementary Table S1.

This paper describes a method that is also based on thermal strain imaging but wherein the temperature is measured directly, in about one hundred milliseconds. The new method provides a quasi-instantaneous measurement of temperature, does not require tracking and cumulating small temperatures changes, and is based on the relation between thermal strain and temperature when using short and constant acoustic power bursts. In this case, the relation is expected to be linear. 

## 2. Principle of the Method

**Figure 1:**
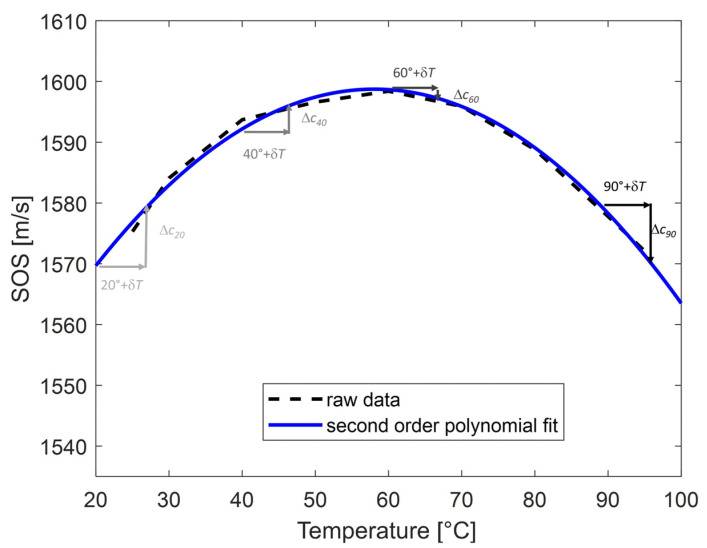
Speed of sound vs. temperature in canine liver samples at 5 MHz (data from [17]). The solid blue line represents the second-order polynomial *f*(*T*) = *a*_0_ + *a*_1_ *T* + *a*_2_ *T*^2^ fitted over the raw data (dashed line). The coefficients of the polynomial are *a*_0_ = 1530 m/s, *a*_1_ = 2.33 m/s/°C, and *a*_2_ = −0.0200 m/s/°C2. The process is illustrated at various temperatures that were chosen arbitrarily (20, 40, 60, and 90 °C). At each temperature, the additional heat pulse increases the temperature by the same amount *δT* (horizontal arrow). The resulting variation in speed of sound Δ*c* is shown by the vertical arrow. The illustration shows that Δ*c* is positive for temperatures below the peak of the parabola, and negative for temperatures above that peak, with a zero crossing at the peak of the parabola. The corresponding thermal strain *s =* −Δ*c*/*c* will therefore exhibit a linear relation with temperature (mathematical justification is given below in the text).

The key to the new method is to change the temperature to be measured temporarily and by a small amount. For this purpose, we used a pulsed heat source designed to induce a local, reproducible, and temporary change of temperature *δT* in the tissue being investigated (Figure 1).

### 2.1. Estimation of Temperature with Known δT and Parabolic Law

For a small *δT*, the change in speed of sound Δ*c* is approximately equal to *δT.∂c*/*∂T*, where *∂c*/*∂T* is the local value of the partial derivative at temperature T. In most biological tissues, the speed of sound can be approximated by a parabolic law:

c(T) ≈ a_0_ + a_1_T + a_2_ T^2^, (1)

when the temperature is varied in the range of 20–100 °C. Under that assumption, the partial derivative *∂c*/*∂T* is a linear function of temperature *T*:

∂c/∂T = a_1_ + 2a_2_T, (2)

The change in speed of sound Δ*c* is therefore also proportional to the temperature:

Δc ≈ δT.∂c/∂T = a_1_δT + 2a_2_δT.T, (3)

As a consequence, the thermal strain *s* induced by this pulsed heat source is also directly proportional to the temperature:

s ≈ −Δc/c = (a_1_ + 2a_2_ T).δT/c, (4)

By rearranging Equation (4), we obtain:

T ≈ −(sc/δT + a_1_)/(2a_2_), (5)

Moreover, in the case of a parabola, as shown in Figure 1, we can introduce *T_c_max_*, which is the temperature that corresponds to the maximum speed of sound (or, in case of fat, to its minimum). It is also the temperature where the derivative of the parabola equals zero, hence:

T_c_max_ = −a_1_/2a_2_, (6)

Equation (5) can then be written as:

T ≈ T_c_max_ − (c/2a_2_δT)s, (7)

Equation (7) has a problem because it includes *c*, the speed of sound, and *c* actually depends on the temperature *T* that we are trying to determine. However, the data available in the literature suggest that the changes in the speed of sound for temperatures between 37 and 100 °C are small, typically ~2% of the average value. Hence, we can replace *c* by its average value *c*_0_:

T ≈ T_c_max_ − (c_0_/2a_2_δT)s, (8)

In the case of the data in Figure 1, for temperatures between 37 and 80 °C, using the average speed of sound *c*_0_ = 1494 m/s instead of the exact value adds an uncertainty of 0.3% on the speed of sound. This uncertainty only induces a small uncertainty of 0.3% on the temperature estimated. Hence, unlike the conventional method presented previously, this new method makes no assumption on the initial temperature and does not require knowledge of the exact speed of sound. The estimation of the temperature is then simply based on the measured thermal strain (Equation (5)).

### 2.2. Estimation of Temperature with Unknown δT 

However, when *δT* is unknown, we can still determine the linear relation experimentally by using two points. Typically, the first point (*T*1 = *T_ref_*, *s*1 = *s_ref_*) would be the stretching factor *s_ref_* measured at body temperature, or at room temperature *T_ref_*. The second point (*T*_2_ = *T_c_max_*, *s*_2_ = 0) can be taken from the literature using the peak of the parabola because by definition, the stretching factor is equal to zero at temperature *T_c_max_*. Combining these two points with Equation (8) yields:

T = T_c_max_ − (T_c_max_ − T_ref_)s/s_ref_, (9)

In this article, the heat source was a HIFU transducer, which is driven by a short acoustic burst that lasts around 100 milliseconds. The measurement is taken within milliseconds after the acoustic burst, so the thermal diffusion and perfusion can be neglected. 

Moreover, in the conventional thermal strain method, the contribution of the thermal expansion on the echo-shift is significant when the temperature is above 50 °C [11,16]:

s = ε − (c(T + δT) − c(T))/c(T),(10)

where *ε* is the thermal expansion, *c* is the speed of sound, *T* is the temperature, and *δT* is the change in temperature. However, in our case, for a small change of temperature *δT*, the thermal expansion induced by that small change was small and could be neglected:

s ≈ −(c(T + δT) − c(T))/c(T),(11)

## 3. Materials and Methods

### 3.1. Simulation

The method relies on the ability of the pulsed heat source to deliver a reproducible variation *δT*. We envisioned that using a HIFU transducer driven at a constant acoustic power and a constant exposure duration would achieve high reproducibility. First, heat source simulations were performed to assess how reproducible this strategy is, especially when the background temperature changes. Then, imaging simulations were performed to assess the feasibility of the method. All simulations included a convergence study.

#### 3.1.1. Simulation: Heat Source

The heat source was a bowl-shaped therapeutic transducer with a diameter of 30 mm, focused at 80 mm, and sinusoidal excitation with frequency of 4.3 MHz (Table 1).

Two-dimensional (2D) ultrasound wave propagation simulations were first conducted at different temperatures using the k-Wave MATLAB toolbox (http://www.k-wave.org, accessed on 17 October 2022) [25,26,27,28]. The model geometry used for the simulations was composed of a homogeneous canine liver matrix (100 mm × 26 mm), and the material properties of the propagation medium were set at a density of 1050 kg/m^3^ and an attenuation coefficient of 0.7 dB/MHz/cm [17,19,29]. To simplify the simulations, the attenuation coefficient was the same for each temperature studied and considered as independent of temperature, as shown for the canine liver tissues by Techavipoo et al. (2002) [17]. Even though other studies have shown the dependence of this property to temperature [17,18], we decided in this study to first explore an ideal case. The speed of sound was defined for each temperature studied using the calibration table (speed of sound vs. temperature in the canine liver samples) from Techavipoo et al. (2002) [17], with the addition of a second-order polynomial fitted over the data.

**Figure 2:**
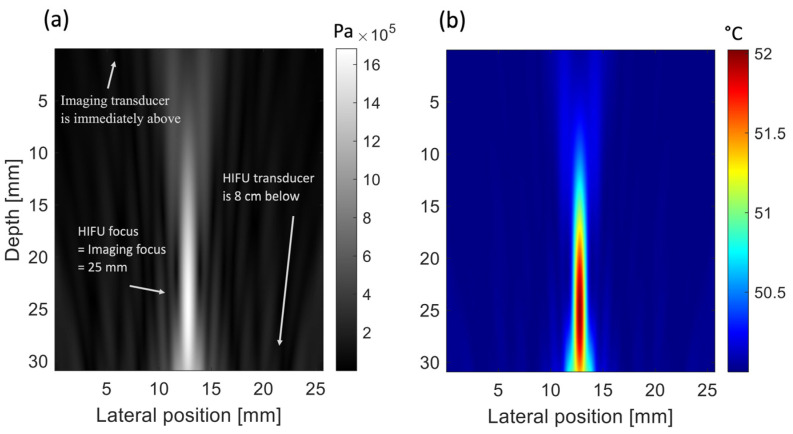
Simulated pressure distribution in a canine liver model. (**a**) Pressure field of the HIFU heat source. (**b**) Corresponding temperature distribution, immediately after 135 ms heating, in a medium at 50 °C.

The coefficients of the polynomial were *a*_0_ = 1531.2 m/s, *a*_1_ = 2.33 m/s/°C, and *a*_2_ = −0.02 m/s/°C2 (Figure 1). From the therapeutic simulations, the pressure amplitude was extracted at each position of the map (Figure 2a). Then, the heat quantity *Q* was calculated at each pixel with the following equation:

Q = αI = αp^2^/2*ρ*c, (12)

where *I* is the acoustic intensity, *α* is the absorption in Neper/m, *p* is the peak acoustic pressure, *ρ* is the density of the medium, and *c* is the speed of sound of the medium at each position of the map.

Simulations of heat diffusion in the homogenous medium were performed using the bioheat transfer equation (BHTE) implemented in k-Wave. This had an initial homogeneous temperature distribution from 20 to 90 °C, a density of 1050 kg/m^3^, a thermal conductivity of 0.512 W/m/K, and a specific heat of 3600 J/kg/K [29]. To create a local increase of 2 °C of the temperature at the focus position, the pressure at the surface of the transducer was set to 2.6 MPa, and the exposure duration was set to 135 ms. 

Simulations were conducted at various background temperature levels (20, 30, 40, 50, 60, 70, 80, and 90 °C). The speed of sound was changed using the data shown in Figure 1. The other parameters were assumed to be constant.

#### 3.1.2. Simulation: Imaging

**Figure 3:**
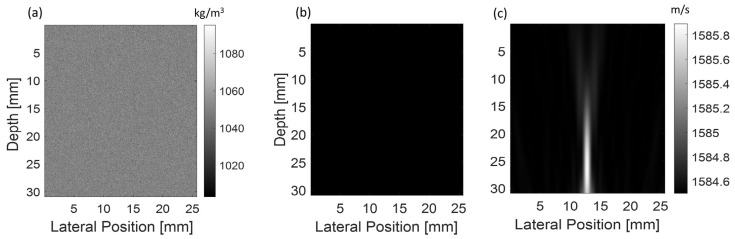
(**a**) Density map for the pre-heating and post-heating simulation. (**b**) Speed of sound map for the pre-heating simulation and (**c**) for the post-heating simulation.

The transmit/receiver linear array of the acoustic imaging simulations represents an L7-4 transducer (Verasonics Inc, Kirkland, WA, USA) configured with 64 elements to speed up the computation of the simulations (element size: 0.25 mm). The elements were excited individually with a broadband pulse (frequency: 5 MHz, bandwidth: 80%, pressure at the surface: 1 MPa, cycle: 2.5) focused at 25 mm. The imaging transducer was placed opposite to the therapeutic transducer. The figures present only a small portion of interest (315 mm × 26 mm) within the simulation region (Figure 2). The focus of the therapeutic and imaging simulations was set at the same position. The density map was identical for the pre- and post-heating simulations (Figure 3a) and defined by changing the density at each point of the matrix with a random Gaussian distribution (i.e., *ρ* = 1050 ± 40 kg/m^3^). The purpose was to create local changes in impedance to generate backscatter. For the pre-heating simulations, the speed of sound map (Figure 3b) was homogenous and set according to the temperature studied (from 20 to 90 °C). For the post-heating simulations, the speed of sound (Figure 3c) was calculated based on the temperature maps of the heat diffusion simulations (Figure 2b) using the fitted calibration table (Figure 1).

Prior to thermal strain computation and temperature estimation, the k-Wave raw data were processed with several post-processing steps. First, the raw RF signals were beamformed (delay and sum) to obtain a single scan line, based on the transducer focus distance and apodization settings. The loss of energy due to attenuation was compensated using time gain compensation, and a Gaussian filter was applied to reduce the noise outside the transmit frequency range [25].

The background temperature of the medium (to be estimated) was homogeneous. For each temperature level, a total of 60 simulations were performed to evaluate the variability induced by the echo-shift estimation during the post-processing. The speckle pattern was varied by changing the density distribution, as explained previously. No random noise was added.

#### 3.1.3. Signal Processing Used in Simulated Data

The time delay between the pre- and post-heating data was calculated using a cross-correlation technique [15,16] and converted to a displacement. In order to achieve high accuracy, a two-step implementation of the zero-lag cross correlation method was used [30]. In the first step, a coarse estimation was obtained using two adjacent windows (2 mm length each, 0% overlap) centered around the focus position. This coarse estimation is an integer number of temporal samples. This was then used to shift and “re-align” the windows. The residual lag (±0.5 time sample) was estimated in the second step using cross-correlation again, with the same window length and overlap, but only testing for three delays (−1, 0, and +1 time sample). Three-point cosine interpolation was then used to estimate the residual delay with sub-sample accuracy [31]. The final time delay estimate was the sum of the coarse estimate and the residual estimate. In addition, amplitude modulation correction (AMC) was also used to improve the estimation accuracy [32]. AMC is designed to compensate for the bias on time-delay estimates, which is induced by intra-window variations in RF signal amplitude. AMC bias correction is based on an accurate estimation of the center of mass of the displacement of the windows. Finally, the thermal strain was obtained from the gradient of the displacement in the vertical (depth) direction.

Two measurement points were used to serve as calibration, assuming a linear relation between the thermal strain and temperature, then Equation (9) was used to determine the temperature. The first was at the initial temperature (*T*_1_ = *T_ref_* = 20 °C; *s*_1_ = *s_ref_*), and the second was at the maximum temperature reached by the parabola (*T*_2_ = *T_c_max_* = 60 °C; *s*_2_ = 0) (Figure 1). The other measurement points (thermal strain from 30 to 90 °C) were then converted to temperature using this calibration and compared to the ground truth values.

### 3.2. Experiments

#### 3.2.1. Experimental Setup

An L7-4 linear array transducer connected to a Verasonics Vantage system (Vantage 256, Verasonics., Kirkland, WA, USA) was used to acquire the beamformed RF signals pre- and post-heating. The details of the imaging transducer are described in Table 1. A custom therapeutic transducer with a PZT ceramic from Boston piezo-optics (Boston piezo-optics, Bellingham, MA, USA) connected to a generator and an amplifier were used to create a burst (time: 150 ms, power: 20 W) and locally increase the temperature at the focus position. The details of the therapeutic transducer are reported in Table 1.

**Figure 4:**
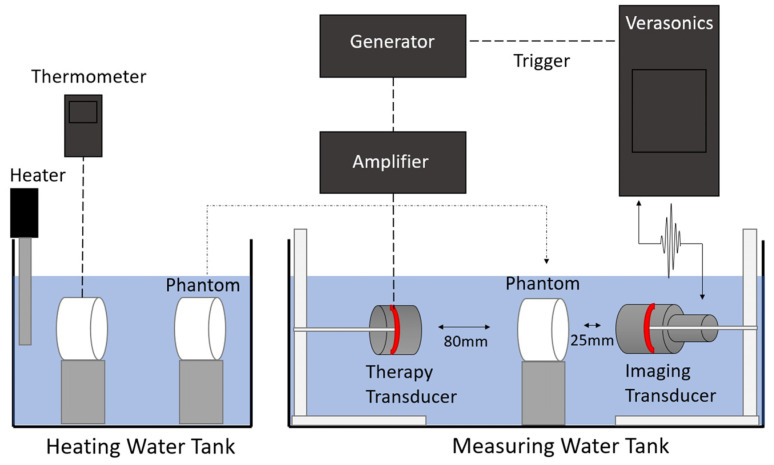
Illustration of the experimental setup. The sample temperature was controlled by setting the water bath temperature at the desired level. The therapy transducer was only used to deliver the small additional heat pulse. For temperatures up to 40 °C, only one water tank was used. For temperatures 50 °C and above, two water tanks were used, one to heat the samples (tank on the left) and one maintained at 37 °C to perform the measurements (tank on the right), because the transducers would have been damaged otherwise. Images were acquired with an L7-4 transducer connected to a Verasonics Vantage system.

The imaging transducer and therapy transducer were positioned opposite each other (Figure 4), and the beam imaging technique [33] was used to align them. Briefly, the beam imaging technique consists of using a conventional ultrasound scanner with no transmitted signal and simultaneous reception on all elements of the probe to capture and display the therapeutic ultrasound beam in real-time.

For temperatures of 23 up to 40 °C, a homogeneous phantom cylinder of Zerdine, tissue-mimicking material (CIRS Inc., Norfolk, VA, USA) was placed at the focal positions of both transducers and imaged in a water tank at different temperatures (23, 25, 30, and 40 °C). A second identical phantom sample with a K-type thermocouple (diameter of 0.2 mm) inserted with a hollow needle was used to control the temperature during the measurements. 

For temperatures of 50 °C and above, the two samples were immerged and heated in a second water tank, then both transferred to the first water tank. We waited for 30–60 s to let the water waves settle down, then both measurements (ultrasound and thermocouple) were recorded simultaneously. 

The first method (one bath) was preferred because it induced a highly controlled and homogeneous temperature distribution within the samples. However, for temperatures >40 °C, the imaging probe would have been damaged. This is why the second bath was added, allowing us to test samples at 50 °C and above at the cost of a less controlled temperature.

At each temperature, the measurements were repeated three times. Between the three repetitions, both samples were transferred back to the heating tank, and we waited for the temperature to stabilize again. Then, the small and temporary increase in temperature was induced by the therapeutic transducer. A 12-angle plane wave imaging sequence was used to acquire 120 frames (total duration 1.5 s) [34]. The time between angles was 500 µs, and the resulting frame rate was 80 fps.

#### 3.2.2. Signal Processing Used in Experiments

The time delays were calculated between the image acquired before the burst (image 5) and the images following the burst (images 20 to 120) by two-step correlation and amplitude modulation corrections, exactly as described in the post-processing simulation section [15,16,32]. The cross-correlations were used along the RF signals, starting 1-mm from the imaging transducer, with a window length of 1 mm and no spacing between the nonoverlapping windows, creating a map of time delays. This time delay map was converted to a displacement map, then filtered with a 3 × 3 pixel (0.9 × 3 mm) moving average filter to reduce the noise. Undesirable sample displacement and deformation were present. These were induced by water movement during the transfer from one tank to another and by HIFU exposure. This undesirable deformation was estimated in two regions outside the focus and subtracted from the displacement map. The thermal strain map was obtained using the gradient of the filtered displacement map, and a second moving average filter (3 × 3 pixels, 0.9 × 3 mm) was applied to further reduce the noise. The thermal strains at the focus were then extracted from the map. 

Two measurement points were used to serve as calibration, assuming a linear relation between the thermal strain and temperature. The first was at the initial temperature (*T*_1_ = *T_ref_* = 23 °C; *s*_1_ = *s_ref_*) and the second (*T*_2_ = *T_c_max_*; *s*_2_ = 0) was the thermal strain equal to zero (where *T_c_max_* in Zerdine was estimated from the experimental data). The other measurement points (thermal strain from 30 to 88 °C) were then converted to temperature using this calibration and compared to values obtained from the thermocouple measurements carried out on the phantom replica.

## 4. Results

### 4.1. Simulation Results

**Figure 5:**
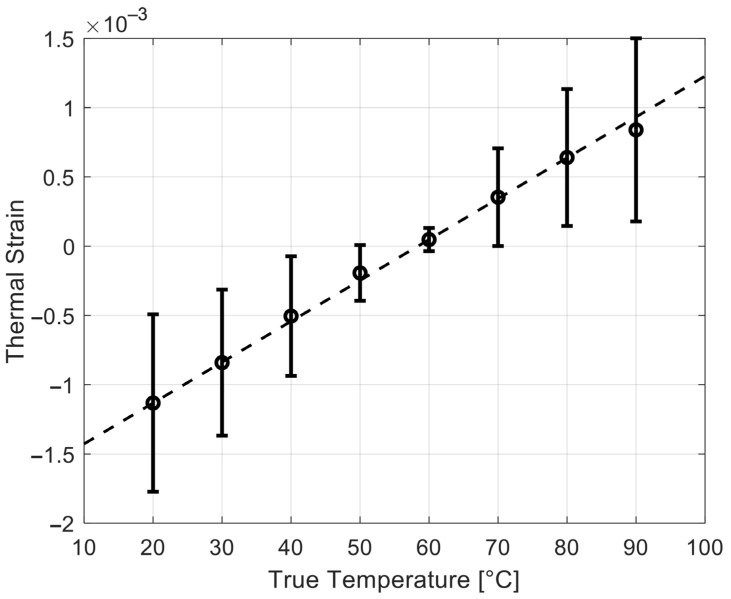
Simulation results of the thermal strain in the canine liver simulations. The error bars represent one standard deviation (n = 60 realizations for each temperature). The dashed line is a linear fit based on the thermal strain values at 20 and 60 °C.

**Figure 6:**
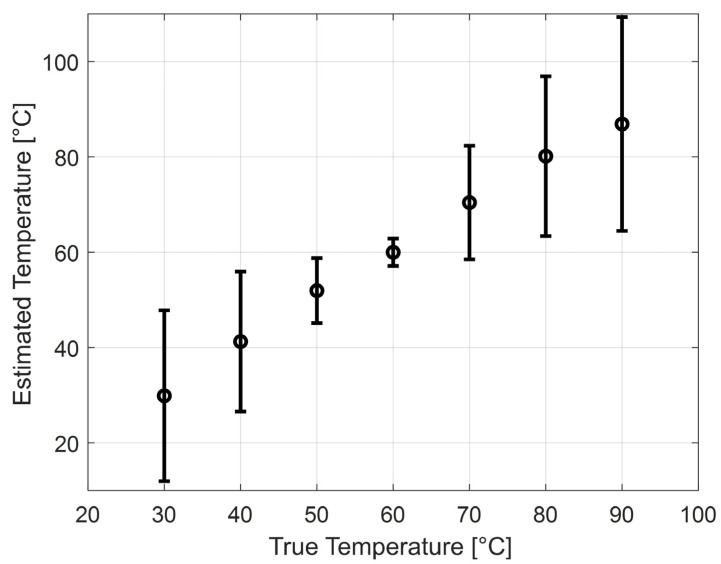
Simulation results of the mean and standard deviation of the estimated temperature using the thermal strain as a function of temperature. The conversion from thermal strain to temperature was performed using the thermal strains previously simulated at 20 and 60 °C and a linear fit (Figure 5) for the 60 canine liver simulations at each temperature step (from 30 to 90 °C).

Figure 5 shows the thermal strain vs. temperature in the canine liver simulations (n = 60 for each temperature). The dashed line is the linear fit based on thermal strain values at 20 and 60 °C. This linear relation was used in Figure 6 to convert the other thermal strain values (i.e., those obtained from 30 to 90 °C) to the temperature. Figure 6 shows the corresponding temperatures, estimated from the thermal strains and the linear fit.

### 4.2. Experimental Results

**Figure 7:**
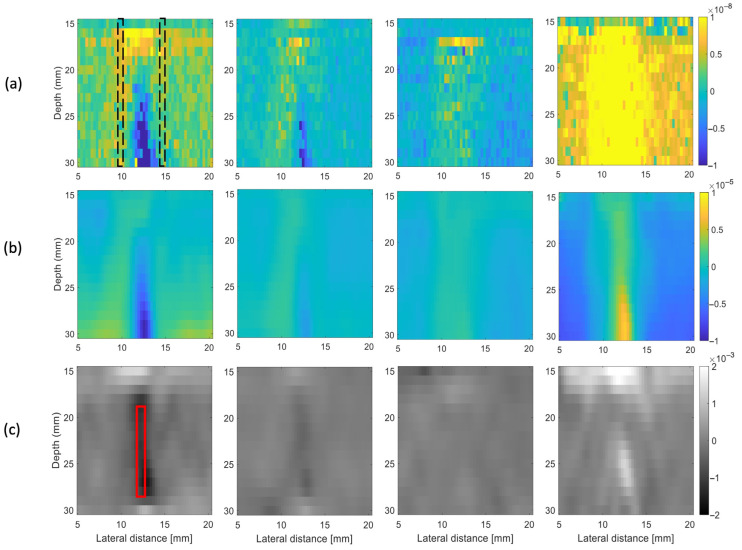
Post-processing steps of data acquired from the experimental measurements for the samples measured at 23, 40, 60, and 80 °C. (**a**) The first row represents the delay maps (in units of seconds) of the 24th frame. The dashed black boxes represent the region used to estimate the undesirable deformations. (**b**) The second row represents the displacement maps (in meters) after an average filter and subtraction of the undesirable deformation. (**c**) The last row represents the thermal strain maps after spatial averaging. The thermal strain is dimensionless and computed as the spatial gradient in the vertical direction of the displacement maps. The red square represents the focus position where the thermal strains were extracted.

Figure 7a shows the time delay maps computed on the 24th frame at 23, 40, 60, and 80 °C. Figure 7b shows the corresponding displacement maps after smoothing and subtraction of the undesirable deformation (black dashed boxes). Figure 7c shows the corresponding thermal strain images after smoothing, along with the focus position where the thermal strains were extracted and spatially averaged (10 × 0.6 mm) (red square).

**Figure 8:**
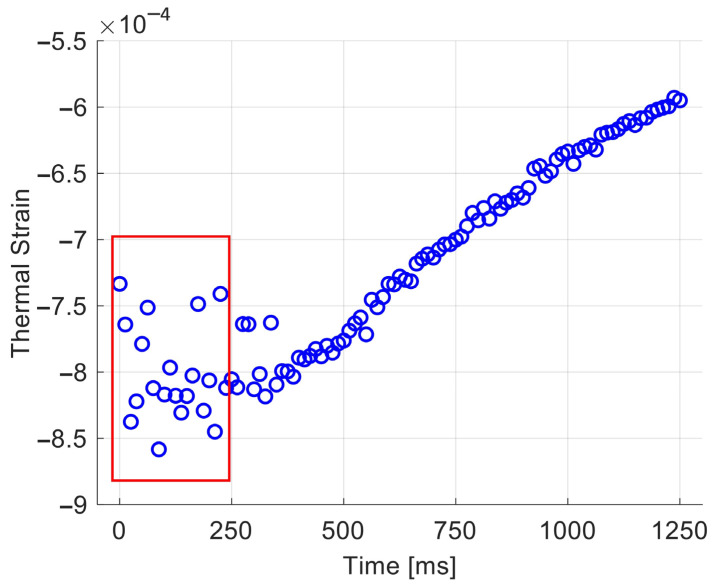
Spatially-averaged thermal strains (Frames 20–120) extracted from the map (Figure 7c) at the focus position for the sample measured at 23 °C. The origin of time was set to frame #20 (i.e., immediately after the end of the burst). The red box indicates the frames where an averaging was applied. The high variability in that time window is unexplained and may be attributable to motion induced by radiation force during HIFU exposure. After that transient regime (i.e., for t > 250 ms), the thermal strain showed lower variability, but it slowly decayed toward zero, resulting in a time-dependent bias. This behavior was attributed to cooling of the heat pulse.

The process was repeated for every image frame, resulting in a time-varying spatially-averaged thermal strain value (Figure 8). The final (spatially-averaged and temporally averaged) thermal strain value for the corresponding experiment was calculated by averaging over the 20 first frames after the burst (red square in Figure 8). After that transient regime (i.e., for t > 250 ms), the thermal strain showed lower variability, but it slowly decayed toward zero, resulting in a time-dependent bias. This behavior was attributed to cooling of the heat pulse.

**Figure 9:**
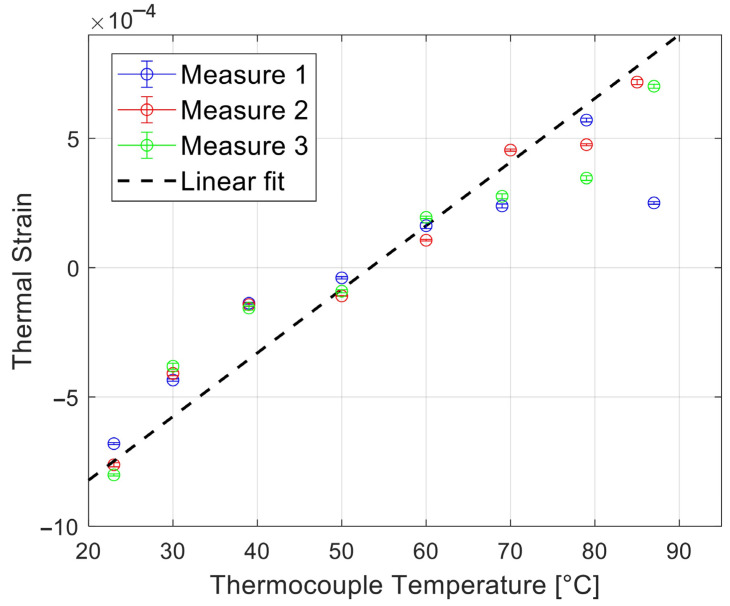
Experimental results of the final (spatially and temporally averaged) thermal strains, calculated for the three measurements at different temperatures from 23 to 88 °C. The dashed line represents a linear fit based on the thermal strains at 23 and at 53.5 °C only.

**Figure 10:**
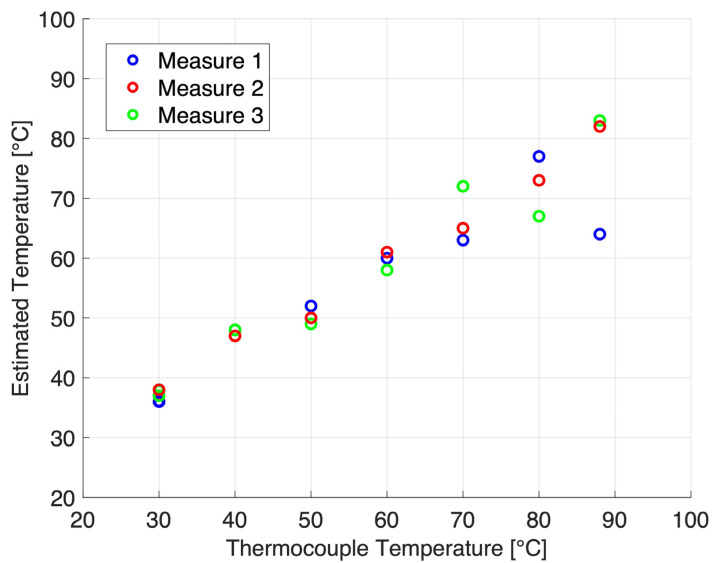
Estimated temperature using the thermal strain as a function of temperature. The conversion from thermal strain to temperature was based on the thermal strain values already shown in Figure 9. The linear fit (dashed line in Figure 9) was used to convert from the thermal strain values to temperature.

Figure 9 shows the thermal strains for temperatures from 23 to 88 °C, along with a linear fit (dashed line) based on the thermal strain values at 23 °C (initial temperature) and 53.5 °C (zero-thermal strain). Figure 10 shows the corresponding temperature estimates in the range of 30–88 °C.

## 5. Discussion

Thermal strains induced by a constant acoustic power bust was estimated in the canine liver simulations and experimentally in the Zerdine phantom samples at different temperatures. The thermal strains were derived from echo-shifts on beamformed RF data, generated by a small and local change in temperature. The thermal strain was then directly converted to estimate the temperature by using a calibration table built from the thermal strain at the initial temperature (20 °C) and the zero-thermal strain (around 60 °C). This calibration enabled us to estimate the temperature without knowing the *δT*, and enables a possible use of the new method in vivo. The results, both in the simulations and experiments, demonstrated the linear relation between thermal stain and temperature and the feasibility of estimating temperature over a wider range of temperatures compared to previous studies [11,17,18,35,36]. 

In this study, the reproducibility of the heat source was obtained by using the same exposure duration and acoustic power by positioning the phantom sample at the same distance of the therapeutic transducer and by assuming constant attenuation, acoustic intensity, specific heat, and density for every measurement, as shown for some tissues in previous studies [17,37]. However, during a typical HIFU treatment, these parameters are likely to vary with temperature [29], especially when coagulation occurs [17,18,37,38]. 

### 5.1. The Case of Coagulation

During coagulation, most tissues exhibit an increase in acoustic attenuation [17,18]. This is why we anticipate that the temperature measurements can become erratic once coagulation has occurred. If this increase in attenuation occurs on the propagation path, it will result in less energy at the focus, and the thermal strain will tend toward zero because of *δ*T being significantly less than expected. On the other hand, if the increase in attenuation occurs at the focus, it will result in higher absorption of the heating pulse, and the thermal strain will depart from zero more than expected because of *δ*T also being higher than expected. The two effects might coexist, making it difficult to establish a general relation.

As a consequence, we anticipate that it will not be possible to measure the temperature in regions where coagulation has occurred. However, it may not be an issue. Indeed, in most therapeutic applications, detecting coagulation is more important than measuring the temperature inside the coagulated area. Interestingly, the temperature T_c_max_, for which the thermal strain becomes zero, also corresponds to the temperature at which coagulation occurs almost instantly, regardless of the duration of exposure. As a consequence, to ensure that coagulation has occurred, we anticipate that it might be sufficient to deposit heat locally until the local thermal strain becomes zero. However, further work is needed to confirm this hypothesis.

### 5.2. The Case of Non-Constant Acoustic Attenuation

Even in the absence of coagulation, the attenuation may change with temperature on the propagation path or at the focus. We anticipate that our thermometry method can only be used in the temperature range wherein the attenuation does not change significantly.

This temperature range depends on the type of tissues. Techavipoo et al. (2004) showed non-constant acoustic attenuation for canine muscle, kidney, and prostate in the temperature range from 25 °C to 95 °C [17]. The case of canine liver was unclear, with nearly constant attenuation reported in 2002 [17], and varying attenuation reported in 2004 [18]. 

Nevertheless, and in spite of these limitations, the case of liver is worth investigating. Indeed, radiofrequency (RF) ablation is the most common procedure for liver cancer and liver metastases. However, the treatment is limited by the lack of feedback regarding the temperature distribution. If attenuation turns out to be constant, our thermometry method has the potential to provide temperature feedback during the procedure.

### 5.3. The Case of Tissue Heterogeneity

Additionally, the reproducibility of the heat source can also be affected by the heterogeneity of the biological tissue being treated or by different biological tissues present on the propagation path.

In the first case (i.e., when the target itself is heterogenous), this difference in composition will have an impact on the parameters considered constant (specific heat, acoustic attenuation, etc.), so the temporary change of temperature δT will not be the same from one region to another. The two-point calibration method will actually take care of this condition. Indeed, for the first measurement, the thermal strain is measured at body temperature directly inside the target, just before starting the treatment. However, the second point (T_c_max_) may change because of the heterogeneity of the tissue, resulting in erroneous measurements.

In the second case (i.e., for heterogeneous tissues present along the acoustic path), the local heating δT cannot be predicted. However, the two-point calibration method is still applicable.

### 5.4. Other Limitations

In order to use the technique in vivo, additional steps will be needed. We envision that it will be necessary to establish calibration tables, such as the ones presented here (Figure 9), for each tissue of interest. The two points necessary for the construction of these calibration tables can be determined in vivo using a thermocouple or an MRI as temperature measurement references, ensuring its applicability in clinics. In fact, nowadays, thermal therapies are screened by ultrasound or MRI, and to build the calibration tables, the second point (T_c_max_) can be measured in vivo during treatment by using a thermocouple or directly by MRI during the procedure.

Future applications in vivo will require heat pulses with shorter durations, typically less than 50–70 ms, to minimize the interframe decorrelation due to undesirable tissue motion [39]. In this work, we used a low power amplifier (20 W), hence the pulse duration needed to induce a detectable thermal strain was about twice that value (150 ms). In future studies, shorter pulses can easily be achieved using higher power amplifiers (50 W or more). Nevertheless, the method appears promising because it might be implemented in conventional ultrasound scanners by using the same imaging probe to induce the heat pulse and perform imaging. The heat pulse is low enough (<2 °C) to be compatible with diagnostic scanning (thermal index <2). In a diagnostic setting, repeated measurements are possible, but the frame rate will have to be limited in order to prevent thermal buildup and possible burns. On the other hand, in therapeutic applications, the frame rate can be increased, and the heat pulse will contribute to the therapeutic effect. 

Other limitations of the study must be mentioned. The region of interests on the simulation data and experimental data were not exactly same. For the simulations, echo-shifts were extracted only from the central scan line on two consecutive windows at the focal position. The size of the matrix and the calculation power of our computer did not allow us to carry out simulations of full B-mode images. In the experiments, the entire focal region was used to extract a spatially-averaged thermal strain (10 × 0.6 mm), but was also temporally averaged (20 frames, 250 ms) to compensate for this course estimation. As mentioned in the Methods section, for experimental measurements above >40 °C, we transferred the phantom from one tank to another to preserve the imaging probes from heat-induced damage. This transfer could have caused a temperature gradient in the phantom due to heat transfer. Moreover, at temperatures above 70 °C, the experimental measurements were challenging. The sample holder started melting and no longer held the phantom in place, which caused the inconsistency of the measurements at these temperatures.

## 6. Conclusions

This study demonstrated that the new ultrasound-based method is a promising technique to obtain a linear relation between the thermal strain and temperature as well as a promising method to estimate the temperature before denaturation of the sample. Future studies should focus on the estimation of temperature in biological tissues, and then on the monitoring of treatment during HIFU therapy and RF ablation.

## 7. Patents

There is a patent resulting from the work reported in this manuscript and protects this invention. European Patent Application number EP24305756.9.

## Figures and Tables

**Table 1:** Acoustic and geometric properties of the transducers used.

Transducer	Imaging	Therapy
Frequency (MHz)	5	4.3
Number of elements	128 (experiment) 64 (simulation)	1
Size pitch (mm)	0.25	n/a
Diameter (mm)	n/a	30
Focus distance (mm)	25	80
Heat pulse duration (ms)	n/a	150 (experiment) 35 (simulation)

## Data Availability

Data will be made available upon reasonable request.

## Supplementary Materials

The following supporting information can be downloaded at: https://www.mdpi.com/article/10.3390/s25020385/s1, Table S1: Summary of the main ultrasound-based methods to estimate temperature.
