# Supplementary material for: A Novel Ultrasound Thermometry Method Based on Thermal Strain and Short and Constant Acoustic Bursts: Preliminary Study in Phantoms
Source: Sensors (Basel). 2025 Jan 10;25(2):385. doi: 10.3390/s25020385 (PMC11769532; doi:10.3390/s25020385)
Supplement: Supplementary file 1 [file sensors-25-00385-s001.zip › sensors-3330680-supplementary.pdf]

Table S1. Summary of the main ultrasound-based methods to estimate temperature.

Method	References	Type	Validation	Temperature range
BSE	[1,8,22]	Relative	In-vivo	<60 °C
TS	[1,8,11,20,21]	Relative	In-vivo	<50 °C
ATT	[1,23,24]	Relative	In-vivo	<50 °C

BSE: Backscattered energy, TS: Thermal strain, ATT: Attenuation
